# Point-of-care ultrasound for general practitioners: a systematic needs assessment

**DOI:** 10.1080/02813432.2020.1711572

**Published:** 2020-01-20

**Authors:** Thomas Løkkegaard, Tobias Todsen, Leizl Joy Nayahangan, Camilla Aakjaer Andersen, Martin Bach Jensen, Lars Konge

**Affiliations:** a Copenhagen Academy for Medical Education and Simulation, University of Copenhagen and The Capital Region of Denmark, Copenhagen, Denmark;; b Research Unit for General Practice in Aalborg Department of Clinical Medicine, Aalborg University, Aalborg, Denmark;; c Department of Otorhinolaryngology, Head and Neck Surgery and Audiology, Rigshospitalet, University of Copenhagen, Copenhagen, Denmark

**Keywords:** Point-of-care testing, primary care, Delphi study, curriculum, ultrasonography, general practice

## Abstract

**Objective:** The aim of the study was to achieve consensus among a group of ultrasound proficient general practitioners (GPs) from Denmark, Norway, Sweden and Finland on which ultrasound scanning modalities and ultrasound-guided procedures are essential to GPs in their daily work for the purpose of including them in a basic ultrasound curriculum.

**Design:** The Delphi methodology was used to obtain consensus.

**Subjects:** Sixty Scandinavian GPs with more than two years of point-of-care ultrasound (POCUS) experience were invited to join the Delphi expert panel.

**Main outcome measures:** In the first Delphi round each member of the panel was asked to produce a list of scanning modalities and procedures which they found relevant to include in a basic ultrasound curriculum. In Delphi round two, these suggestions were presented to the entire panel who assessed whether they found them essential in their daily work. Items not reaching consensus in round two, were presented to the panel in a third and final round. Items reaching more than 67% agreement were included.

**Results:** Forty-five GPs were included in the study and 41 GPs completed all rounds. Agreement was obtained on 30 scanning modalities and procedures primarily within the musculoskeletal (8), abdominal (5), obstetric (5) and soft tissue (3) diagnostic areas. Four ultrasound-guided procedures were also agreed upon.

**Conclusion:** A prioritized list of 30 scanning modalities and procedures was agreed upon by a group of ultrasound proficient GPs. This list could serve as a guideline when planning future POCUS educational activities for GPs.

**Key points**
- Point-of-care ultrasound (POCUS) is increasingly being used by general practitioners (GPs), but little is known about which ultrasound applications are most used.
- We performed a systematic needs assessment among a group of ultrasound proficient GPs using the Delphi methodology for the purpose of establishing a basic POCUS curriculum.
- The process resulted in a prioritized list of 30 scanning modalities and ultrasound guided procedures.
- Our study provides the basis for an evidence-based basic POCUS curriculum for GPs.

## Introduction

In recent years the use of point-of-care ultrasound (POCUS) has gained ground in general practice in part facilitated by the development of compact, low-cost, high-quality ultrasound scanners [1]. In addition to this, a new generation of general practitioners (GPs) are expected to incorporate POCUS in patient care after having acquired ultrasound competencies during their pre- and postgraduate training [2,3]. Hence, the use of POCUS in general practice is assumed to increase significantly in the coming years.

POCUS examinations are different from traditional comprehensive ultrasound examinations which cover an anatomical region, assess more than one organ and result in a full report of the examination. Instead, POCUS examinations are performed to achieve specific procedural aims (e.g. direct the needle to the correct location) or answer focused clinical questions (e.g. ‘does my patient have ascites?’) [4,5].

Ultrasonography is a user dependent image modality and competence is needed to ensure diagnostic accuracy [6]. POCUS requires a combination of anatomical and clinical knowledge, technical skills and the ability to interpret ultrasound images [7]. Failure to provide GPs with the necessary skills could lead to false positive findings, eliciting unnecessary patient anxiety and further redundant testing. Similarly, false negative findings could lead to potentially dangerous diagnoses being overlooked. Acquiring and sustaining competencies of various scanning modalities and procedures require training and continual exposure to relevant clinical conditions.

Many scanning modalities could be included in a basic POCUS curriculum for general practitioners [8], and several ultrasound curricula have been suggested and incorporated into general practice residency training programs [9–13]. While many studies have demonstrated that GPs are able to perform various ultrasound scanning protocols [14–17], few studies have addressed which competencies are relevant in general practice. Further, none of the curricula published have been evidence-based or prioritized. Therefore, a systematic needs assessment to guide a POCUS curriculum specific to general practice is called for.

The aim of the study was to achieve consensus among a group of ultrasound proficient GPs from Denmark, Norway, Sweden, and Finland on which ultrasound scanning modalities and procedures are essential to GPs in their daily work for the purpose of including them in a basic ultrasound curriculum.

## Methods

### Study design

A Delphi methodology was used to conduct a systematic general needs assessment to explore which ultrasound scanning modalities and procedures are essential to GPs in their daily work in order to establish a basic ultrasound curriculum [18,19]. The Delphi technique entails setting up a panel of experts in an area of interest. Panelists are anonymous to each other thereby avoiding dominant individuals to interfere with and unduly influence the process. The process consists of multiple rounds. In the first round, a brainstorm is performed by each participant in order to establish a comprehensive pool of suggestions for the panel to evaluate. In consecutive rounds, participants rate the items in order to come to an agreement on which items should be included in the end result. Rounds are iterated until consensus has been reached on some or all of the items. Usually, three rounds suffice, but more rounds can be added if agreement has not been reached. We set the level of agreement to two-thirds majority (67%) which is widely accepted in the literature [20].

### Selection of Delphi panel members

The following inclusion criteria were used: participants had to be GPs, work in a permanent position in a general practice in Denmark, Sweden, Norway or Finland, have completed a basic ultrasonography course which included basic physics, ‘knobology’ and an introduction to more than two scanning modalities and have used POCUS on a daily basis for more than two years. Potential participants were excluded if they had conflicts of interest (e.g. financial) or had a colleague in the same clinic who was already participating in the study. Key members of national ultrasound societies (Danish Society for Ultrasound in General Practice (DAUS) and Association for Ultrasound in General Practice (FUA Norway) were identified and asked to provide names of potential participants. Since no formal ultrasound societies for general practitioners exist in Sweden and Finland, participants were recruited through informal interest groups and course providers. Back-ground information about the study was provided to the participants by means of a homepage where the complete protocol was published (www.gp-ultrasound.com). Invitations were sent by e-mail. If potential participants met the inclusion criteria, they were asked to provide informed consent. The participants were anonymous to each other.

### The Delphi process

The data collection was conducted from September 2018 to January 2019.

#### Delphi round 1: brainstorm

After inclusion each panel member received an e-mail including instructions on how to complete the questionnaire. The participants were asked to provide information about themselves, practice characteristics, POCUS use and equipment availability.

The panel members were asked the following question: ‘which scanning modalities and/or procedures should be included in a basic ultrasound curriculum for general practitioners? i.e. which modalities/procedures should every GP with ultrasound equipment be able to perform?’. A list of all the replies were produced and content analysis was applied by the project group (TL, CAA and MBJ) in order to allow for identification of items that were either too non-specific, similar or could be grouped in the same category. First, replies were categorized according to overall scanning area, e.g. musculoskeletal, abdominal and gynecological. Secondly, according to organ, e.g. kidney, bladder and uterus, and lastly according to condition, e.g. gall stone, living intrauterine pregnancy or abdominal aortic aneurism. If scanning protocols were suggested, they were subdivided into their individual constituent parts in order to avoid misunderstandings due to participants having different perceptions of the content of the protocol, e.g. FATE: pericardial effusion, chamber dimensions, wall thickness or estimation of ejection fraction. In case of difficulties categorizing an item, the project group resolved the problem by group discussion until consensus was reached. Items suggested by only one participant were excluded.

#### Delphi round 2: rating of scanning modalities

Panelists received an e-mail with instructions on how to fill out the questionnaire and a link to the second round of the survey. For each item, they were presented with the following question: ‘this scanning modality/procedure is essential for my work as a general practitioner’ and had to rate the statement on a five-point Likert scale where five was ‘strongly agree’ and one was ‘strongly disagree’.

An item was included in the final list of scanning modalities and procedures if it reached agreement (Likert score ≥ 4) by more than two-thirds (≥67%) of the participants. If the item reached agreement by less than one-third (≤33%) of the participants, it was excluded. Remaining items would go on to a third round for re-evaluation.

#### Delphi round 3: re-prioritization and elimination

In round three panelists were asked to re-rate the remaining items in accordance with the question in round two. To facilitate the responses, panelists were provided with a list of their previous replies and the overall results of the replies from the panel. Items reaching more than two-thirds agreement (≥67%) were included in the final list. The rest were excluded.

### Statistics

Participant characteristics were recorded as categorical and continuous variables and characterized by descriptive statistics accordingly. Medians, ranges, frequencies, percentages and cumulative percentages were calculated for each item in round two and three. Consensus level was set to 67%. Mean Likert score for each item was calculated together with the standard deviation in order to rank each item in order of importance. Pearson´s correlation coefficient was calculated in order to explore correlation between agreement levels in round two and three.

The questionnaires were sent out using SurveyMonkey^®^ and statistical analysis of data was performed with the IBM^®^ SPSS^®^ software package, version 25.0 (SPSS, Chicago, IL).

### Ethics

Ethical approval was granted in the form of an exemption letter from the Regional Ethical Committee of the Capital Region, Denmark (file-number: H-18017138). Participants granted informed consent by replying to the invitation.

## Results

**Figure 1.**
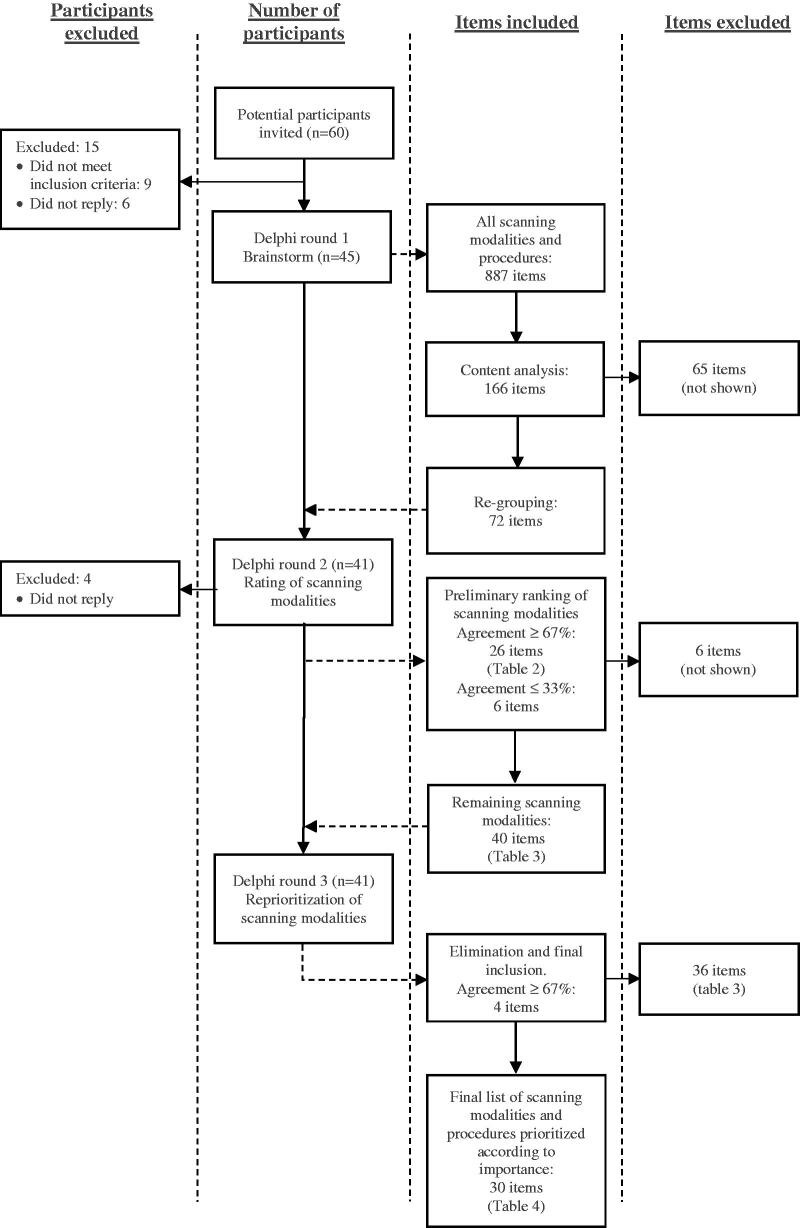
Study flow. Items: scanning modalities and procedures. *N*:number of panelists in the study.

Forty-five (75%) of the 60 general practitioners invited were included in the survey (Figure 1). Nine did not meet the inclusion criteria and six did not reply to the invitation. Of the 45 panelists who replied in round one, 41 participated in round two (91%) and all 41 participated in round three (100%). Characteristics of the panelists completing round three are summarized in Table 1.

**Table 1.** Delphi panelist characteristics (*n* = 41).

Mean age (SD)	50 (9)
Gender	
Male	32
Female	9
Country	
Denmark	24
Norway	10
Sweden	6
Finland	1
Ultrasound experience (years)	
2–3 years	15
4–5 years	10
>5 years	16
Employment status	
Owner	37
Employee	4
Practice location	
Rural (most of the patients live in rural areas)	7
Urban (most of the patients live in urban area)	18
Mixed (patients live in both urban and rural areas)	16
Distance to nearest radiological facility where ultrasound services are provided	
<10 km	25
10–50 km	11
>50 km	5
Number of doctors working in practice	
1	5
2–3	19
4–5	10
>5	7
Average number of ultrasound examinations performed daily	
<1	6
1–2	17
3–4	14
>4	4
Ultrasound teaching experience	
Yes	19
No	22
On-call duty (medical emergency services)	
Yes	21
No	20
Use of POCUS during on-call duties	
Yes	17
No	4
Transducers used	
Curve-linear (abdominal)	40
Linear	37
Gynecological	30
Phased array (cardiac)	21
3D/4D	4
Hockey stick	2
Type(s) of scanning modalities and procedures performed	
Abdominal	39
Urinary tract	38
Obstetrics	36
Abdominal aorta	36
Musculoskeletal	33
Gynecology	32
Softtissue (lumps and bumps)	31
Deep venous thrombosis	29
Ultrasound guided injections	25
Testis	23
Lung	22
Cardiac	19
Thyroid	16
Carotid	12

The brainstorm process in round one resulted in 887 suggestions of scanning modalities and ultrasound-guided procedures in all. Content analysis including removal of duplicates reduced the number of items to 166. After elimination of items suggested by only one panelist and further re-grouping, the total number of items which were carried through to round two was 72 (Figure 1).

In round two, 26 items were agreed upon by more than 67% of the panelist qualifying these items to be included in the final list without further re-evaluation (Table 2). Six items were excluded since less than 33% agreed to these. Forty items went on to round three (Table 3). In round three, four items: rotator cuff tendinitis and/or ruptures (partial/full), injection/aspiration knee joint, elbow joint effusion and trochanter bursitis, increased their level of agreement, and were added to the final list of items. A Pearson´s correlation coefficient of 0.78 between agreements in rounds two and three was obtained. Table 4 shows all the items included in the final list prioritized according to their mean Likert score. The most common scanning modalities were within the musculoskeletal (8), abdominal (5), obstetric (5) and soft-tissue (3) diagnostic areas. Additionally, four ultrasound-guided procedures were included: shoulder and knee injection, Bakers cyst injection/aspiration and ultrasound guided abscess drainage.

**Table 2.** List of scanning modalities and procedures which reached agreement in round 2 (≥67%).

	Agreement	Median Likert score	Range	Mean Likert score	SD
Bladder volume	100%	5	4–5	4.71	0.46
Subcutaneous abscesses	98%	5	3–5	4.49	0.55
Gall stones	98%	5	3–5	4.71	0.51
Hydronephrosis	95%	5	2–5	4.44	0.74
Localization of intrauterine device	95%	5	2–5	4.51	0.68
Living intrauterine pregnancy	93%	5	2–5	4.61	0.77
Fetal position	93%	5	2–5	4.54	0.71
Cholecystitis	93%	5	2–5	4.44	0.78
Localization of foreign body	85%	4	2–5	4.27	0.78
Free abdominal fluid	85%	5	3–5	4.49	0.75
Achilles tendinitis and tendon rupture	85%	4	1–5	4.29	0.87
Bakers cyst	85%	5	1–5	4.37	0.89
Abdominal aortic aneurism	85%	5	2–5	4.41	0.81
First trimester bleeding	85%	5	2–5	4.39	0.86
Deep venous thrombosis	83%	5	2–5	4.37	0.83
Subcutaneous tumors (lipoma, atheroma)	83%	4	2–5	4.27	0.81
Gestational age (CRL measurement)	80%	5	2–5	4.32	1.01
Pericardial effusion	78%	4	2–5	4.10	0.92
Pleural effusion	76%	4	2–5	4.20	0.93
Subacromial/subdeltoid bursitis	76%	4	2–5	4.00	0.95
Ultrasound guided abscess drainage	73%	4	1–5	3.90	1.02
Injection shoulder	73%	4	2–5	4.20	0.90
Varicocele/hydrocele	73%	4	1–5	3.80	1.05
Knee joint effusion	73%	4	3–5	4.20	0.84
Biceps tendinitis, tenosynovitis, and tendon rupture	71%	4	1–5	4.00	0.97
Injection/aspiration, Bakers cyst	68%	4	15	3.95	1.02

**Table 3.** List of scanning modalities and procedures in round 2, which were carried through to round 3 by level of agreement (percent).

	Agreement^a^	Median Likert score	Range	Mean Likert score	SD
Pneumothorax	66% (63%)	4	1–5	3.88	1.00
Extrauterine pregnancy	66% (61%)	4	2–5	3.63	1.04
Ganglion	66% (66%)	4	1–5	3.98	1.01
Injection/aspiration knee joint^b^	66% (68%)	4	2–5	4.00	1.00
Rotator cuff tendinitis and/or ruptures (partial or full)^b^	66% (78%)	4	1–5	3.85	1.15
Trochanter bursitis^b^	63% (68%)	4	1–5	3.63	1.07
Kidney and bladder stones	63% (46%)	4	1–5	3.68	1.08
Fasciitis plantaris	61% (66%)	4	1–5	3.63	0.97
Injection, acromioclavicular joint	61% (61%)	4	2–5	3.85	0.91
Patellar ligament tendinitis	59% (61%)	4	1–5	3.56	1.14
Testicular tumors	56% (37%)	4	1–5	3.46	1.25
Elbow joint effusion^b^	56% (68%)	4	1–5	3.51	1.19
Injection, fascia plantaris	56% (54%)	4	1–5	3.63	1.13
Interstitial syndrome (presence of b-lines)	56% (54%)	4	1–5	3.68	1.17
Splenomegaly	56% (39%)	4	1–5	3.37	1.30
Lateral and medial epicondylitis	56% (44%)	4	1–5	3.51	1.00
Uterine fibromas	54% (41%)	4	2–5	3.51	0.84
Injection, epicondylitis (lateral and medial)	51% (46%)	4	1–5	3.66	1.09
Vein puncture (blood sampling)	51% (39%)	4	1–5	3.66	0.94
Estimation of ejection fraction	51% (39%)	4	1–5	3.46	1.14
Pneumonia	51% (44%)	4	1–5	3.54	1.00
Ovarian cysts	51% (46%)	4	2–5	3.54	1.05
Fetal growth (Biparietal diameter, abdominal and head circumference and femur length)	49% (34%)	3	1–5	3.22	1.26
Childhood obstipation (rectal diameter)	49% (46%)	3	1–5	3.51	1.14
Arthrosis acromioclavicular joint	49% (54%)	3	1–5	3.51	1.16
Fractures of long bones (e.g. clavicle, metatarsals, metacarpals, phalanges) and costae	49% (61%)	3	2–5	3.49	0.87
Prostate hypertrophy	46% (29%)	3	1–5	3.32	1.06
Assessment of wall thickness	46% (29%)	3	1–5	3.22	1.13
Mammary cysts	44% (41%)	3	1–5	3.24	1.07
De Quervain tendinitis	44% (41%)	3	1–5	3.32	0.96
Assessment of chamber dimensions	44% (29%)	3	1–5	3.24	1.16
Placenta previa	44% (34%)	3	1–5	3.24	1.16
Hepatomegaly	44% (24%)	3	1–5	3.02	1.17
Thyroid cysts	41% (39%)	3	1–5	3.27	1.12
Bladder Tumor	41% (27%)	3	1–5	3.17	1.34
Hernia	41% (49%)	3	1–5	3.12	1.03
Hip joint effusion	37% (39%)	3	1–5	3.27	1.16
Carotid stenosis	37% (27%)	3	1–5	3.10	1.07
Liver metastases	34% (39%)	3	1–5	2.88	1.42
Quadriceps tendinitis	34% (24%)	3	1–5	3.15	0.85

Note: Agreement in round 3 is shown in parenthesis.

a Pearson’s correlation coefficient between agreement in round 2 and 3 (*r* = 0.78).

b Items are included in the final prioritized list (Table 4).

**Table 4.** Final prioritized list of scanning modalities and procedures which have gained consensus by level of and importance (mean).

		Mean Likert score	SD	Agreement
1.	Bladder volume	4.71	0.46	100.0%
2.	Gall stones	4.71	0.51	97.6%
3.	Living intrauterine pregnancy	4.61	0.77	92.7%
4.	Fetal position	4.54	0.71	92.7%
5.	Localization of intrauterine device	4.51	0.68	95.1%
6.	Free abdominal fluid	4.49	0.75	85.4%
7.	Subcutaneous abscesses	4.49	0.55	97.6%
8.	Hydronephrosis	4.44	0.74	95.1%
9.	Cholecystitis	4.44	0.78	92.7%
10.	Abdominal aortic aneurism	4.41	0.81	85.4%
11.	First trimester bleeding	4.39	0.86	85.4%
12.	Bakers cyst	4.37	0.89	85.4%
13.	Deep venous thrombosis	4.37	0.83	82.9%
14.	Gestational age (CRL measurement)	4.32	1.01	80.5%
15.	Achilles tendinitis and tendon rupture	4.29	0.87	85.4%
16.	Subcutaneous tumors (lipoma, atheroma)	4.27	0.81	82.9%
17.	Localization of foreign body	4.27	0.78	85.4%
18.	Injection shoulder	4.20	0.90	73.2%
19.	Pleural effusion	4.20	0.93	75.6%
20.	Knee joint effusion	4.20	0.84	73.2%
21.	Pericardial effusion	4.10	0.92	78.0%
22.	Subacromial/subdeltoid bursitis	4.00	0.95	75.6%
23.	Biceps tendinitis, tenosynovitis and tendon rupture	4.00	0.97	70.7%
24.	Injection/aspiration, Bakers cyst	3.95	1.02	68.3%
25.	Rotator cuff tendinitis and/or ruptures (partial or full)	3.93	1.01	78.0%
26.	Ultrasound guided abscess drainage	3.90	1.02	73.2%
27.	Varicocele/hydrocele	3.80	1.05	73.2%
28.	Injection/aspiration knee joint	3.73	1.14	68.3%
29.	Elbow joint effusion	3.71	1.05	68.3%
30.	Trochanter bursitis	3.59	1.05	68.3%

## Discussion

### Principal findings

We conducted a systematic needs assessment using the Delphi method among a group of 41 Scandinavian GPs who have used POCUS daily for more than two years. This process resulted in a list of 30 scanning modalities and procedures which a majority of panelists found essential to their work as GPs and could be considered for inclusion in a basic ultrasound curriculum. Simple scanning modalities and procedures within the musculoskeletal, obstetric, abdominal and soft tissue diagnostic areas were most prevalent.

### Strengths and weaknesses of the study

The strength of our study is that we succeeded in establishing a homogenous panel of experienced GPs in enough numbers to obtain a valid result [21]. A low drop-out rate between rounds was achieved indicating a dedicated and motivated panel. Panelists characteristics were representative of a diverse population of GPs and they provided many scanning modalities and procedures in round one, which secured a robust and representative selection of scanning modalities to be assessed in rounds two and three.

Our study has some limitations. GPs from each country were not represented in equal numbers. Panelists were mainly male and primarily worked in urban practices relatively close to radiological services (<50 km) thereby limiting the generalizability of our results. GPs in rural settings and in remote areas might find more advanced scanning modalities like lung and cardiac POCUS applications more useful than GPs working in an urban setting where easy access to emergency services and radiological departments is possible. Furthermore, primary care in the Scandinavian countries share many similarities regarding funding, access, visitation and referral to other specialties and the secondary sector [22]. This may limit the relevance of our findings to countries where primary care does not play the same central role.

Even though consensus was reached on some items, there were also significant disagreements which is reflected in the wide intervals in range and standard deviation for many of the items. This divergence of opinions among panelists could be due to differences in gender, national guidelines, ultrasound experience, special interests, work requirements and geography. Our study was not designed to explore if significant differences between subgroups were present, however post-hoc statistical analysis indicated that Danish GPs were more restrictive regarding which scanning modalities and procedures to include in a basic curriculum (data not shown).

We acknowledge some methodological limitations regarding the way we designed the Delphi process which may have affected the validity of our findings. The condensation procedure excluded many items, which consequently did not have a chance to be rated and included in the final list. We eliminated items from round three which had gained consensus or were only agreed upon by less than a third in round two to secure a manageable number of items for the panelists to rate in round three. This contributed to a high response rate but did not allow for the panelists to rate the items in round three again.

### Findings in relation to other studies

To our knowledge this is the first time an evidence-based systematic needs assessment of ultrasound scanning modalities for the purpose of establishing a basic POCUS curriculum for office-based GPs has been published. The American Academy of Family Practitioners (AAFP) has published a curriculum guideline for family medicine residents which proposes a wide range of POCUS applications, many of which are also included in our study [9]. Applications are divided into basic and advanced according to consensus opinion. However, the guideline does not state how consensus has been reached and among whom. The authors suggest that successful implementation requires that course providers decide which modalities they find most useful in their setting. Our study is an attempt to provide an evidence-based basic POCUS curriculum for GPs in accordance with the AAFP guideline recommendation.

Most POCUS curricula for GPs published are based on assumptions about which modalities are useful for GPs, developed to be used in other settings than general practice, e.g. emergency departments or designed by general practitioners affiliated with academic centers [10–13]. In addition to this, these ultrasound curricula bear considerable resemblance to curricula published for emergency physicians, which limit their relevance to the average GP [23,24].

The most common scanning modalities in our study were within the musculoskeletal, abdominal, obstetric and soft-tissue diagnostic areas. To some extent this is consistent with previously published studies about the use of POCUS in general practice. Thus, Andersen et al. [8] found that abdominal and obstetric scanning modalities were most reported in the literature.

However, there are also substantial differences. Panelists in our study prioritized musculoskeletal scanning modalities which made out most of the scanning modalities. Given that musculoskeletal symptoms are among the most common presentations in general practice this is not surprising [25]. However, only few studies have been conducted of both training in and use of musculoskeletal ultrasound in general practice [8].

Cardiac and pulmonary scanning modalities, excluding pericardial and pleural effusion, did not gain consensus. These findings were surprising given that several studies suggest that limited cardiac examinations can be mastered by GPs and implemented in clinical practice [16,26,27]. Nearly half of the panelists (49%) did not have phased array transducers and only 46% performed cardiac examinations which may explain their low priority in our study. The limited use of cardiac scanning modalities could be due to the fact that phased array (cardiac) transducers are more costly than curved array (abdominal) transducers. Furthermore, cardiac examinations are time consuming and require considerable training and routine to perform [28,29]. GPs might also omit cardiac examinations in clinical decision making due to the more severe consequences of making an erroneous diagnosis or overlooking significant pathology. A concern that seems well founded in the literature where cardiac examinations have lower diagnostic accuracies than other scanning modalities [8].

Pulmonary scanning modalities did not reach agreement. This might reflect the fact that lung ultrasound is a new scanning modality and not widely utilized yet, even though it has been shown to be useful in general practice [30].

The majority of panelists in our study performed 1–4 ultrasound scans a day which is in accordance with other studies [8]. Legitimate concern could be raised about how to maintain adequate training if POCUS is applied to a wide range of indications. General practice is characterized by low disease prevalence rates and accordingly low predictive values of tests. Thus, scanning patients with high pre-test probabilities of having specific conditions is important to be aware of in order to obtain a correct result. In our study panelists agreed on simple scanning modalities of common clinical conditions, e.g. knee joint effusion and gall stones, which indicate that they recognize this concern.

One might argue that 30 items are a lot to cover in a basic curriculum. However, if a more limited curriculum is wanted the final prioritized list can still aid the course provider in deciding which items are most relevant to include.

### Meaning of the study

Our study offers a proposal for the content of a basic ultrasound curriculum for GPs. The Delphi panel primarily agreed upon simple scanning modalities and procedures mainly within the musculoskeletal, abdominal, obstetric and soft tissue diagnostic areas. Cardiac, pulmonary and more advanced gynecological scanning modalities did not gain consensus and should probably not be included in a basic POCUS curriculum but reserved for more experienced GPs. Our data also shows a considerable range of opinions which need to be considered when establishing a curriculum. Future studies should focus on development of evidence-based educational activities for GPs and residents in primary care medicine.
